# Molecular characterization of ten viral pathogens causing calf diarrhea in Hanwoo (*Bos Taurus coreanae*) by next generation sequencing

**DOI:** 10.1186/s12917-025-04926-2

**Published:** 2025-07-14

**Authors:** Jeong-Byoung Chae, Won Gyeong Kim, Shinae Song, Joon-Seok Chae

**Affiliations:** 1 Laboratory of Veterinary Internal Medicine, BK21 FOUR Future Veterinary Medicine Leading Education and Research Center, Research Institute for Veterinary Science and College of Veterinary Medicine, Seoul National University, Gwanak-ro 1, Gwanak-gu, Seoul, 08826 Republic of Korea; 2 Bio Team, Animal Industry Data Korea, Seoul, 06152 Republic of Korea

**Keywords:** Calf diarrhea, Hanwoo calves, Viral detection, Prevalence, Next-generation sequencing, Phylogenetic analysis

## Abstract

**Background:**

Calf diarrhea remains a significant concern in the global cattle industry, leading to considerable economic losses. Infectious pathogens are among the primary causes of this disease. In this study, the prevalence of 7 pathogens—bovine rotavirus (BRV), bovine coronavirus (BCV), bovine viral diarrhea virus (BVDV) types 1 and 2, *Cryptosporidium parvum*, *Giardia* spp., and *Eimeria* spp.—associated with calf diarrhea was investigated using polymerase chain reaction (PCR). A metagenomic approach was also applied to identify additional RNA viral pathogens from unknown causes of diarrheic fecal samples in the Republic of Korea (ROK).

**Results:**

A total of 810 fecal samples from Hanwoo calves (*Bos taurus coreanae*) were collected, consisting of 526 normal samples (267 with a fecal score of 0 and 259 with a fecal score of 1) and 284 diarrheic samples (178 with a fecal score of 2 and 106 with a fecal score of 3). All 7 pathogens were detected by PCR in feces and their detection rates and mean fecal scores for each were as follows: BRV (14.0%, 1.41), BCV (3.2%, 1.42), BVDV1 (2.1%, 1.35), BVDV2 (4.9%, 1.33), *C*. *parvum* (9.8%, 1.66), *Eimeria* spp. (1.9%, 1.73), and *Giardia* spp. (0.9%, 0.71). Among these pathogens, BRV (*p* = 0.004), *C*. *parvum* (*p* < 0.001), and *Eimeria* spp. (*p* = 0.027) showed an increase in prevalence with higher fecal scores. Twenty-one fecal samples negative for all pathogens were randomly selected and subjected to high-throughput sequencing to identify RNA viral pathogens associated with calf diarrhea. This approach led to the identification of nearly complete genomic sequences for bovine astrovirus, bovine enterovirus, bovine kobuvirus, bovine nebovirus, bovine norovirus, bovine boosepivirus B, bovine parechovirus, bovine torovirus, *C*. *parvum* virus 1, and hunnivirus.

**Conclusions:**

This study represents the first investigation of hunnivirus presence and provides a comprehensive description of the nearly complete genomes of 10 viruses associated with calf diarrhea in the ROK. The findings contribute to a better understanding of the epidemiology and molecular characteristics of calf diarrhea-associated pathogens in the ROK, highlighting the potential application of high-throughput sequencing for diagnosing other diseases.

**Supplementary Information:**

The online version contains supplementary material available at 10.1186/s12917-025-04926-2.

## Background

Calf diarrhea remains a significant concern in the cattle industry, leading to considerable economic losses due to reduced growth and increased morbidity and mortality in neonatal calves [1]. This condition is caused by a variety of factors, including environmental influences, management practices, and infectious agents [2]. Among these, infectious causes have long been recognized as primary contributors to calf diarrhea, with the presence of specific pathogens closely linked to the occurrence of this disease [3]. Historically, major pathogens such as bovine rotavirus (BRV), bovine coronavirus (BCV), bovine viral diarrhea virus (BVDV), *Cryptosporidium parvum*, *Giardia* spp., and *Eimeria* spp. have been consistently associated with calf diarrhea [4–7].

Numerous studies, including those conducted in the Republic of Korea (ROK), have investigated the causative agents of calf diarrhea [7–10]. However, most of these reports have relied on traditional diagnostic methods, particularly polymerase chain reaction (PCR), to target specific pathogens. While traditional diagnostic techniques, such as bacterial and viral cultures, serological assays, and microscopy, are valuable, they are often limited by their inability to detect unculturable or previously unknown pathogens. This limitation has contributed to a significant number of undiagnosed cases and delays in timely treatment, particularly when the etiological agent is novel or emerging.

The metagenomic approach, utilizing next-generation sequencing (NGS), has revolutionized pathogen detection in recent years [11]. This technology, which allows for the sequencing of millions of DNA fragments in a single run, has enabled the detection of a broad range of microorganisms—including viruses, bacteria, fungi, and parasites—without the need for prior knowledge or culturing. It facilitates the identification of multiple pathogens in a single sample, providing a more comprehensive understanding of disease etiology. Using this technology, the diagnosis and molecular characterization of previously undiagnosed pathogens has become possible [12, 13].

This study aimed to investigate the prevalence of infectious pathogens (BRV, BCV, BVDV, *C*. *parvum*, *Giardia* spp., and *Eimeria* spp.) associated with calf diarrhea in the ROK. PCR was employed, alongside a metagenomic approach, to target RNA viral pathogens that had previously been overlooked due to their failure to be recognized as major causative agents.

## Results

### Sample collection

To investigate the prevalence of pathogens associated with calf diarrhea, 810 fecal samples were collected from Hanwoo (*Bos taurus coreanae*) calves across 15 farms in 2022. The samples were classified into 2 groups: 526 normal samples (267 with a fecal score of 0 and 259 with a fecal score of 1) and 284 diarrheic samples (178 with a fecal score of 2 and 106 with a fecal score of 3) according to calf health scoring [14] (Table 1).

**Table 1:** Description of collected fecal samples from 810 Hanwoo calves across 15 farms, categorized by fecal scores

Farm IDs	Locations	No. of Hanwoo calves				
		Fecal scores				
		0	1	2	3	Total
1	Anseong	13	16	7	1	37
2	Anseong	23	13	5	1	42
3	Anseong	18	26	28	26	98
4	Anseong	63	38	20	13	134
5	Yesan	5	13	10	5	33
6	Dangjin	39	44	29	8	120
7	Gongju	23	23	9	6	61
8	Cheongyang	12	13	6	3	34
9	Cheongyang	0	6	8	4	18
10	Nonsan	19	9	9	6	43
11	Buan	22	17	23	19	81
12	Namwon	14	19	12	5	50
13	Bongwha	6	4	1	1	12
14	Changnyeong	2	7	1	1	11
15	Sancheong	8	11	10	7	36
Total		267	259	178	106	810

### Association between fecal scores and each pathogen by PCR detection

The association between fecal scores and the presence of each pathogen was examined in the 810 fecal samples and described according to fecal scores (Table 2). Among the pathogens detected, BRV (14.0%, 113/810) was the most prevalent, followed by *C*. *parvum* (9.8%, 79/810), BVDV2 (4.9%, 40/810), BCV (3.2%, 26/810), BVDV1 (2.1%, 17/810), *Eimeria* spp. (1.9%, 15/810), and *Giardia* spp. (0.9%, 7/810). Of the 7 pathogens, the detection rates of BRV (*p* < 0.01), *C*. *parvum* (*p* < 0.001), and *Eimeria* spp. (*p* < 0.05) increased as fecal scores increased (Table 2).

**Table 2:** Detection rates of 7 pathogens in fecal samples from 810 Hanwoo calves, categorized by fecal scores

Pathogens	Fecal scores (No. of Hanwoo calves, %)					*P* values	
	0 (*n* = 267)	1 (*n* = 259)	2 (*n* = 178)	3 (*n* = 106)	Total (*n* = 810)	Pe	Li
BRV	33 (12.4%)	30 (11.6%)	21 (11.8%)	29 (27.4%)	113 (14.0%)	< 0.001	0.004
BCV	7 (2.6%)	8 (3.1%)	4 (2.2%)	7 (6.6%)	26 (3.2%)	0.189	0.170
BVDV1	3 (1.1%)	8 (3.1%)	3 (1.7%)	3 (2.8%)	17 (2.1%)	0.408	0.414
BVDV2	12 (4.5%)	12 (4.6%)	7 (3.9%)	9 (8.5%)	40 (4.9%)	0.336	0.277
*C*. *parvum*	12 (4.5%)	21 (8.1%)	28 (15.7%)	18 (17.0%)	79 (9.8%)	< 0.001	< 0.001
*Eimeria* spp.	2 (0.7%)	4 (1.5%)	5 (2.8%)	4 (3.8%)	15 (1.9%)	0.174	0.027
*Giardia* spp.	3 (1.1%)	3 (1.2%)	1 (0.6%)	0 (0.0%)	7 (0.9%)	0.662	0.257

**Pe**: Pearson’s chi-square test; **Li**: Linear by linear association; **BRV**: Bovine rotavirus; **BCV**: Bovine coronavirus; **BVDV1**: Bovine viral diarrhea virus type 1; **BVDV2**: Bovine viral diarrhea virus type 2; ***C***. ***parvum***: *Cryptosporidium parvum*

### Detection of viruses by NGS

To identify potential viruses causing calf diarrhea, NGS was performed on a subset of 21 fecal samples with a fecal score of 3 and no detection of the 7 known pathogens. The 21 samples were sequenced, generating 5,801,008–82,117,476 reads per sample (mean: 36,672,189; median: 22,283,010). After quality trimming, all reads with a Q30 score or higher were considered high-quality, with over 95% of the reads meeting this threshold. Assembly was conducted using the rnaviral pipeline of SPAdes, yielding contig sequences ranging from 87 to 65,044 nucleotides (mean: 8,438; median: 3,732) for each sample. Contig sequences underwent Basic Local Alignment Search Tool (BLAST) analysis, producing a total of 3,798 results from all samples. After applying a filter with target virus coverage ≥ 90%, a final count of 178 viral contig sequences was obtained. Among these viral contigs, those associated with cattle or diarrhea were further analyzed. In 18 of the 21 samples, more than one virus was detected. Metagenomic analysis revealed nearly complete sequences of 5 viruses in the *Picornaviridae* family (bovine enterovirus (BEV), bovine kobuvirus (BKoV), bovine boosepivirus (BooV), bovine parechovirus (BParV), and hunnivirus), 2 viruses in the *Caliciviridae* family (bovine nebovirus (BNeV) and bovine norovirus (BNoV)), 1 virus in the Astroviridae (bovine astrovirus (BAstV)), 1 virus in the *Tobaniviridae* family (bovine torovirus (BToV)), and 1 virus in the *Partitiviridae* family (*C*. *parvum* virus 1 (CSpV1)) (Table 3).

**Table 3:** Information on detected viruses identified by next-generation sequencing

Farm IDs	Locations	The list of detected viruses by NGS
1	Anseong	BKoV, BooV
11	Buan	CSpV1
1	Anseong	BKoV, BooV
10	Nonsan	-
10	Nonsan	CSpV1
10	Nonsan	-
10	Nonsan	BAstV, BToV
6	Dangjin	BooV, BAstV, CSpV1
4	Anseong	BKoV, BooV, BParV, BEV, hunnivirus
5	Yesan	BKoV, BooV, hunnivirus
10	Nonsan	BToV
11	Buan	-
2	Anseong	BKoV, BooV, BAstV, BEV
11	Buan	BKoV, BooV, BAstV, CSpV1
4	Anseong	BNoV
15	Sancheong	BooV, BNeV, BNoV
4	Anseong	BKoV, CSpV1, hunnivirus
13	Bongwha	BAstV
13	Bongwha	BooV
6	Dangjin	BKoV, BooV, BAstV
4	Anseong	CSpV1

**BAstV**: Bovine astrovirus; **BEV**: Bovine enterovirus; **BKoV**: Bovine kobuvirus; **BNeV**: Bovine nebovirus; **BNoV**: Bovine norovirus; **BooV**: Bovine boosepivirus; **BParV**: Bovine parechovirus; **BToV**: Bovine torovirus; **CSpV1**: *Cryptosporidium parvum* virus 1; -: Not detected

### Genomic characterization and phylogenetic analysis of bovine enterovirus

**Fig. 1:**
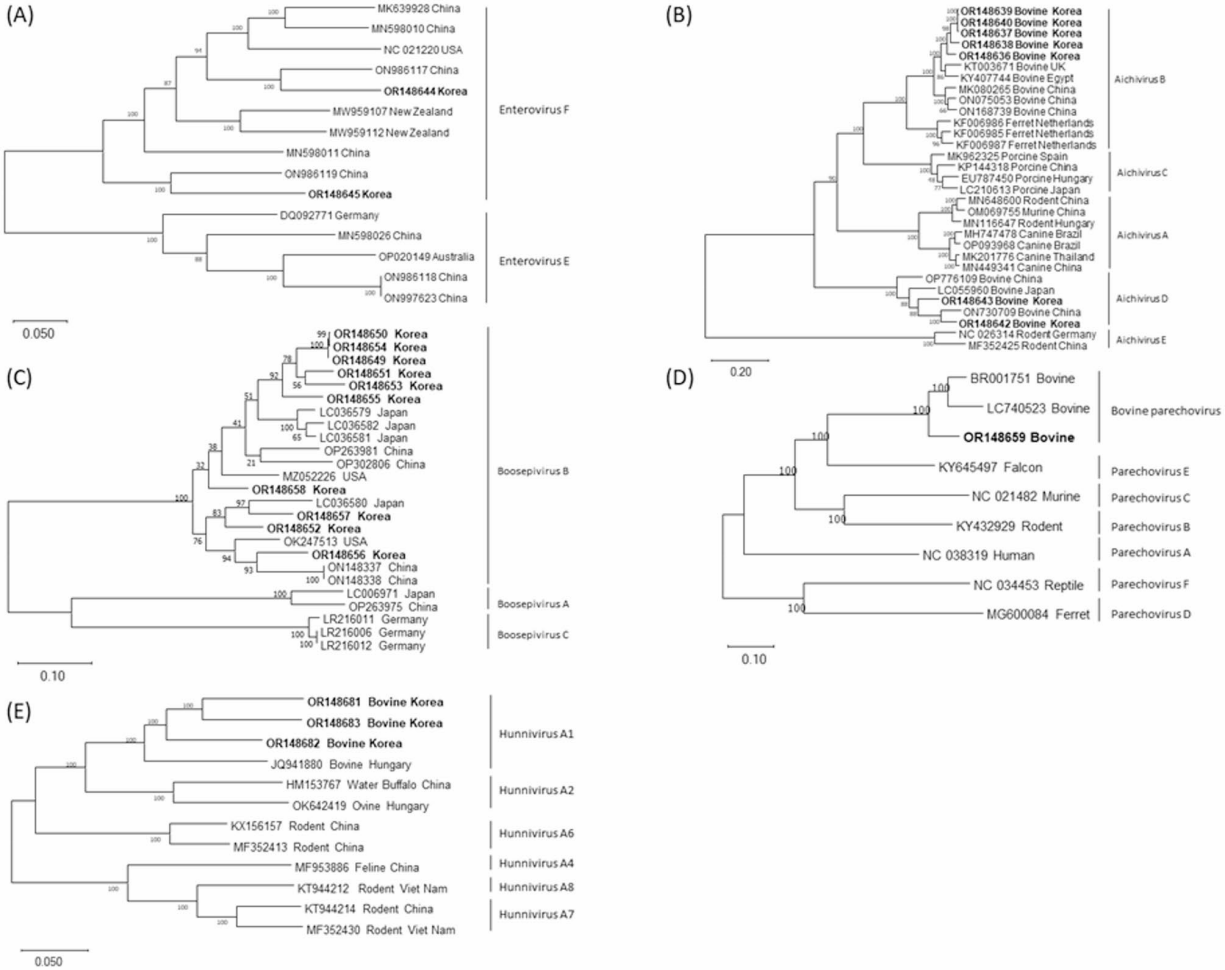
Phylogenetic analysis of 5 *Picornaviridae* viruses identified in Hanwoo calves in this study. [(**A**) Bovine enterovirus (BEV); (**B**) Bovine kobuvirus (BKoV); (**C**) Bovine boosepivirus (BooV); (**D**) Bovine parechovirus (BParV); (**E**) Hunnivirus]. Neighbor-joining phylogenetic trees were constructed based on complete genomic sequences of the obtained sequences in this study. The bar represents genetic distance. Numbers at nodes indicate bootstrap percentages obtained from 1,000 bootstrap replicates. Sequences obtained in this study are marked in bold

Bovine enterovirus (BEV) genomic RNA sequences were identified in 2 of the 21 fecal samples (Table 4), with genomic sizes of 7,365 and 7,398 nucleotides, respectively. Phylogenetic tree analysis based on complete genomic sequences revealed that both samples clustered within the BEV-F strain (Fig. 1A). Homology analysis showed that the 2 obtained sequences exhibited 76.4% and 79.8% nucleotide identity with the BEV-F strain isolate (NC_021220) (Table S4).

### Genomic characterization and phylogenetic analysis of bovine kobuvirus

Bovine kobuvirus (BKoV) genomic RNA sequences were identified in 7 of the 21 fecal samples (Table 4), with genomic sizes ranging from 8,293 to 8,441 nucleotides. Phylogenetic tree analysis based on complete genomic sequences demonstrated that 5 samples belonged to *Aichivirus* B, while 2 samples belonged to *Aichivirus* D (Fig. 1B). Homology analysis revealed that the 5 obtained sequences from *Aichivirus* B showed 89.9–90.2% nucleotide identity with the *Aichivirus* B isolate (KT003671) (Table S5), while the 2 obtained sequences from *Aichivirus* D showed 79.4% and 83.6% nucleotide identity with the *Aichivirus* D isolate (LC055960) (Table S6).

### Genomic characterization and phylogenetic analysis of bovine boosepivirus B

Bovine boosepivirus (BooV) RNA sequences were identified in 10 of the 21 fecal samples (Table 4), with genomic sizes ranging from 7,613 to 7,750 nucleotides. Phylogenetic tree analysis based on complete genomic sequences revealed that all 10 samples clustered within BooV B (Fig. 1C). Homology analysis indicated that the 10 obtained sequences exhibited 83.0-87.3% nucleotide identity with the BooV B isolate (LC036579) (Table S9).

### Genomic characterization and phylogenetic analysis of bovine parechovirus

Bovine parechovirus (BParV) RNA sequence was identified in 1 of the 21 fecal samples (Table 4), with a genomic size of 7,809 nucleotides. Phylogenetic tree analysis based on complete genomic sequences showed that the BParV sequence did not cluster with *Parechovirus* groups A to F but was instead classified with previously reported sequences of other BParV strains (Fig. 1D). Homology analysis revealed that the obtained sequence exhibited 86.3% nucleotide identity with the BParV isolate (BR001751) (Table S10).

### Genomic characterization and phylogenetic analysis of hunnivirus

Hunnivirus genomic RNA sequences were identified in 3 of the 21 fecal samples (Table 4), with genomic sizes ranging from 7,565 to 7,597 nucleotides. Phylogenetic tree analysis showed that all 3 samples clustered with the *Hunnivirus* A1 strain (Fig. 1E). Homology analysis revealed that the 3 obtained sequences exhibited 81.2–83.9% nucleotide identity with the hunnivirus A1 isolate (NC_018668) (Table S14).

### Genomic characterization and phylogenetic analysis of bovine nebovirus

**Fig. 2:**
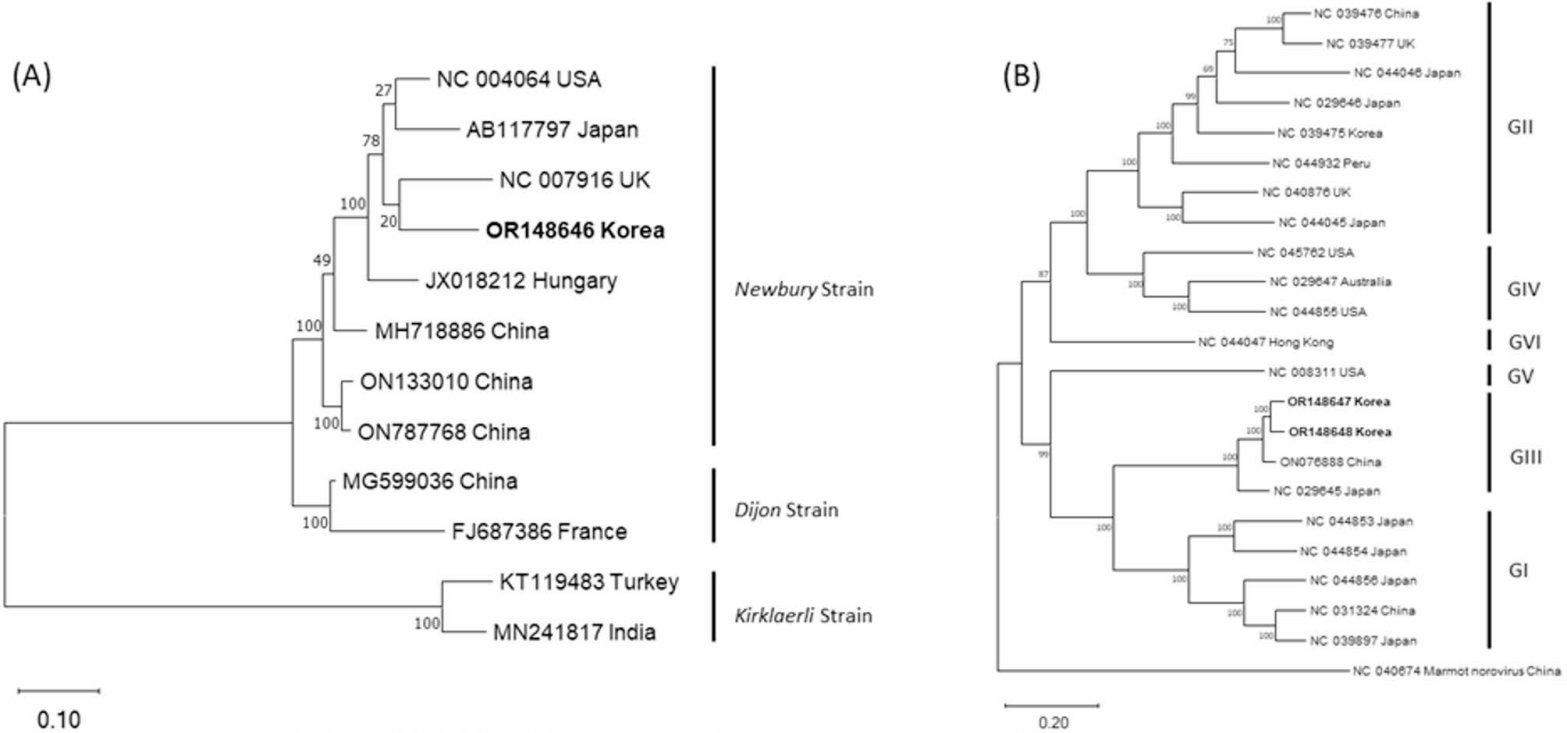
Phylogenetic analysis of 2 *Caliciviridae* viruses identified in Hanwoo calves in this study [(**A**) Bovine nebovirus (BNeV); (**B**) Bovine norovirus (BNoV)]. Neighbor-joining phylogenetic trees were constructed based on complete genomic sequences of the obtained sequences in this study. The bar represents genetic distance. Numbers at nodes indicate bootstrap percentages obtained from 1,000 bootstrap replicates. Sequences obtained in this study are marked in bold

Bovine nebovirus (BNeV) genomic RNA sequence was identified in 1 of the 21 fecal samples (Table 4), with a genomic size of 7,399 nucleotides. Phylogenetic tree analysis showed that this sample clustered within the Newbury strain (Fig. 2A). Homology analysis revealed that the obtained sequence exhibited 81.5% nucleotide identity with the BNeV isolate (NC_007916) (Table S7).

### Genomic characterization and phylogenetic analysis of bovine Norovirus

Bovine norovirus (BNoV) genomic RNA sequences were identified in 2 of the 21 fecal samples (Table 4), with genomic sizes ranging of 7,273 and 7,307 nucleotides, respectively. Phylogenetic tree analysis showed that both samples clustered within the Norovirus GIII type (Fig. 2B). Homology analysis revealed that the 2 obtained sequences exhibited 85.8% nucleotide identity with the Norovirus GIII type isolate (NC_029645) (Table S8).

### Genomic characterization and phylogenetic analysis of bovine astrovirus

**Fig. 3:**
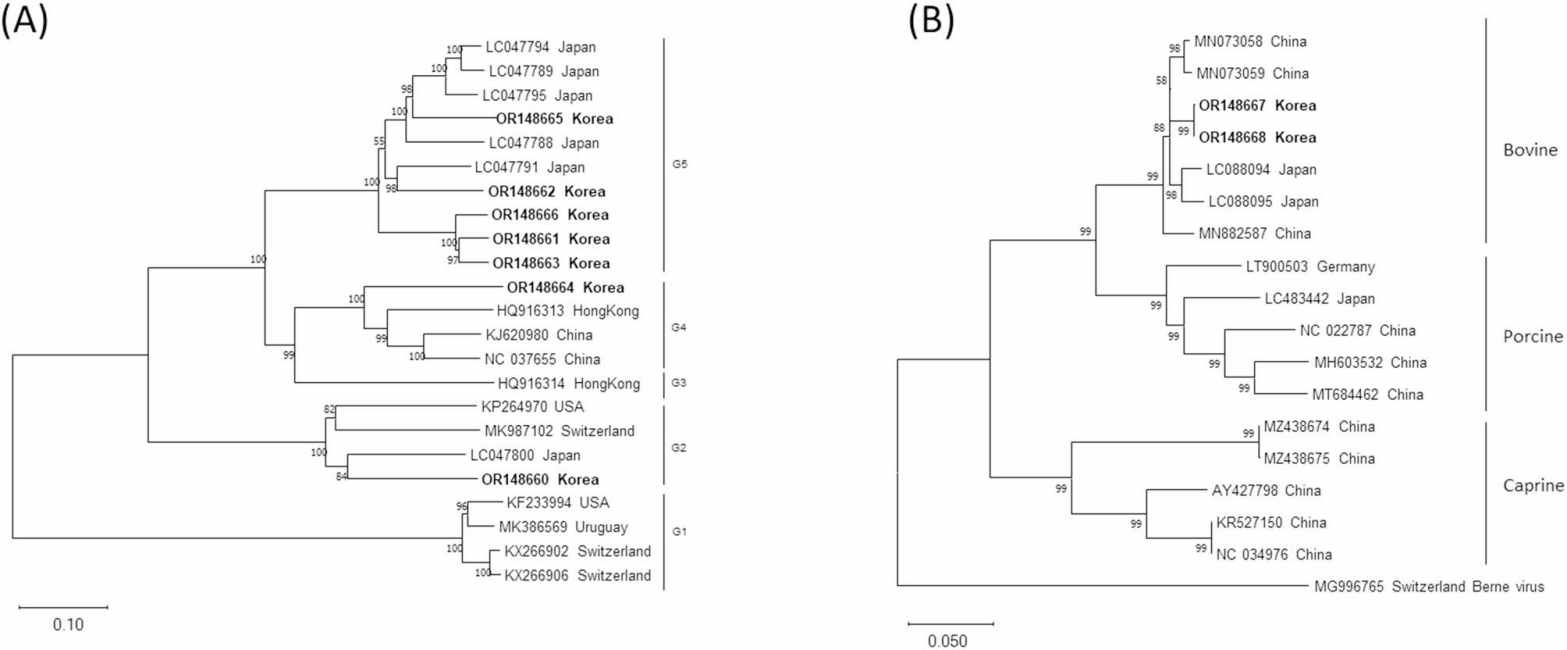
Phylogenetic analysis of (**A**) bovine astrovirus (BAstV) and (**B**) bovine torovirus (BToV). Neighbor-joining phylogenetic trees were constructed based on complete genomic sequences of the obtained sequences in this study. The bar represents genetic distance. Numbers at nodes indicate bootstrap percentages obtained from 1,000 bootstrap replicates. Sequences obtained in this study are marked in bold

Bovine astrovirus (BAstV) RNA sequences were identified in 7 of the 21 fecal samples (Table 4), with genomic sizes ranging from 6,052 to 6,288 nucleotides. Phylogenetic tree analysis based on complete genomic sequences revealed that 1 sample clustered within astrovirus group 2, 1 sample within group 4, and 5 samples within group 5 (Fig. 3A). Homology analysis indicated that the obtained sequence from astrovirus group 2 exhibited 73.8% nucleotide identity with the astrovirus group 2 isolate (LC047800) (Table S1), the obtained sequence from group 4 showed 73.2% nucleotide identity with the group 4 isolate (NC_037655) (Table S2), while the 5 obtained sequences from group 5 demonstrated 77.6–82.9% nucleotide identity with the astrovirus group 5 isolate (LC047788) (Table S3).

### Genomic characterization and phylogenetic analysis of bovine torovirus

Bovine torovirus (BToV) genomic RNA sequences were identified in 2 of the 21 fecal samples (Table 4), with genomic sizes of 27,657 and 28,423 nucleotides, respectively. Phylogenetic tree analysis based on complete genomic sequences showed that both samples were most closely related to the BToV isolate from China (MN073058) but not closely related to the BToV isolate from Canada (AY427798) (Fig. 3B). Homology analysis revealed that the 2 obtained sequences exhibited 80.3% and 82.1% nucleotide identity with the BToV isolates from Canada (AY427798) (Table S11).

### Genomic characterization and phylogenetic analysis of *Cryptosporidium* Parvum virus 1 (CSpV1)

**Fig. 4:**
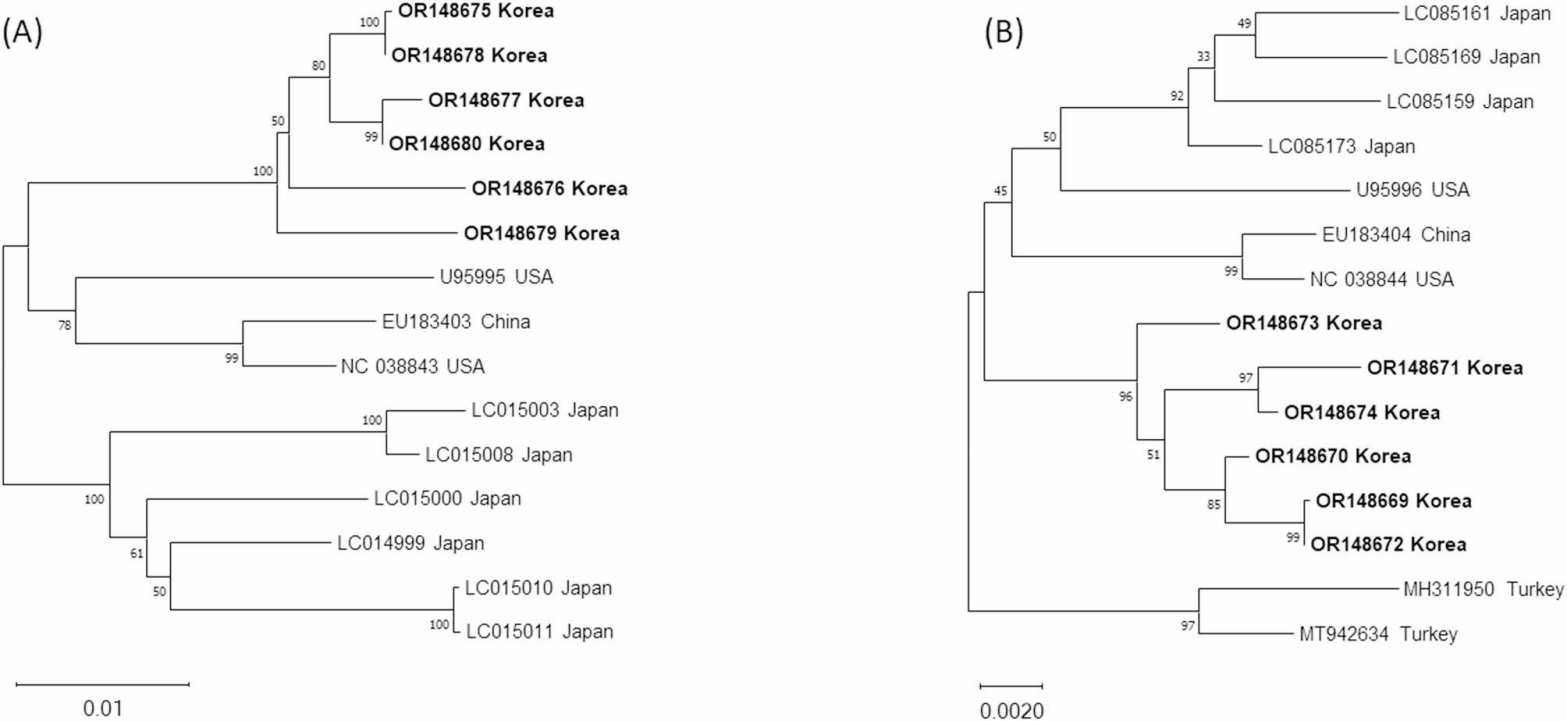
Phylogenetic analysis of *Cryptosporidium parvum* virus 1 (CSpV1) [(**A**) dsRNA1; (**B**) dsRNA2]. Neighbor-joining phylogenetic trees were constructed based on complete genomic sequences of the obtained sequence in this study. The bar represents genetic distance. Numbers at nodes indicate bootstrap percentages obtained from 1,000 bootstrap replicates. Sequences obtained in this study are marked in bold

These viruses contain 2 unrelated, linear dsRNA segments, 1.7 kbp (dsRNA1) and 1.4 kbp (dsRNA2), each separately encapsidated, with dsRNA1 encoding the RNA-dependent RNA polymerase (RdRp) and dsRNA2 encoding the capsid protein. CSpV1 genomic RNA sequences were identified in 6 of the 21 fecal samples (Table 4), with dsRNA1 genomic sizes ranging from 1,721 to 1,853 nucleotides and dsRNA2 genomic sizes ranging from 1,486 to 1,539 nucleotides. Phylogenetic tree analysis based on dsRNA1 and dsRNA2 revealed that the sequences obtained from this study clustered together and were distinct from those reported in other countries (Fig. 4). Homology analysis revealed that the 6 obtained sequences exhibited 95.6–96.1% nucleotide identity with the NCBI reference sequence for CSpV1 dsRNA1 (RdRp) (NC_038843) (Table S12) and 97.8–98.1% nucleotide identity with the NCBI reference sequence for CSpV1 dsRNA2 (capsid protein) (NC_038844) (Table S13).

**Table 4:** Detection rates of viruses associated with diarrhea in 21 Hanwoo calves by next-generation sequencing

Virus	Description of detected viruses	No. of Hanwoo calves (%)
BAstV	BAstV group 2	1 (4.8)
	BAstV group 4	1 (4.8)
	BAstV group 5	5 (24.8)
BEV	*Enterovirus* F	2 (9.5)
BKoV	*Aichivirus* B	5 (24.8)
	*Aichivirus* D	2 (9.5)
BNeV	BNeV Newbury strain	1 (4.8)
BNoV	*Norovirus* GIII	2 (9.5)
BooV	BooV B	10 (47.6)
BParV	-	1 (4.8)
BToV	-	2 (9.5)
CSpV1	-	6 (28.6)
Hunnivirus	*Hunnivirus* A1	3 (14.3)

**BAstV**: Bovine astrovirus; **BEV**: Bovine enterovirus; **BKoV**: Bovine kobuvirus; **BNeV**: Bovine nebovirus; **BNoV**: Bovine norovirus; **BooV**: Bovine boosepivirus; **BParV**: Bovine parechovirus; **BToV**: Bovine torovirus; **CSpV1**: *Cryptosporidium parvum* virus 1; **-**: No classification

## Discussion

Infectious pathogens are a significant cause of diarrhea in calves, and many different pathogens are known to contribute to calf diarrhea [1]. Despite considerable efforts, however, there remains a significant gap in research on the full spectrum of pathogens responsible for calf diarrhea. The application of NGS has proven invaluable in addressing these limitations, advancing both the detection and analysis of pathogens. This study aimed to assess the diagnostic capabilities of NGS by investigating a diverse range of viruses associated with calf diarrhea and to conduct epidemiological investigations of the pathogens that are known to be the primary causes of this condition.

Compared with numerous studies on the prevalence of various pathogens associated with calf diarrhea worldwide [15–17], previous research in the ROK has predominantly focused on major pathogens, including BRV, BCV, BVDV, *Cryptosporidium* parvum, *Giardia* spp., and *Eimeria* species [7, 18, 19]. In this study, BRV was most frequently detected in calf feces, followed by *C*. *parvum*, BVDV2, BCV, BVDV1, *Eimeria* spp., and *Giardia* species. The prevalence of these pathogens exhibited considerable variation, which can be attributed to geographical differences, experimental methodologies, and timeframes. However, the findings of this study were consistent with those of previous investigations, demonstrating no significant discrepancies. Bovine rotavirus, *C*. *parvum*, and *Eimeria* spp. showed a statistically significant association with fecal scores. Both BRV and *C*. *parvum* have been identified as major causes of calf diarrhea, not only in the ROK but also in other countries [7, 20]. Although commercial vaccines for BRV are available, controlling the disease remains challenging. For *C*. *parvum*, no effective vaccine is currently known worldwide. These pathogens continue to present significant challenges, highlighting the need for more comprehensive and targeted research on effective preventive measures. In this study, *Eimeria* spp. showed a significant association with fecal scores; however, the low detection rate limits the reliability of this finding. Future studies are needed to explore the relationship between *Eimeria* spp. and calf diarrhea in greater detail.

In this study, 10 different viruses (BAstV, BEV, BKoV, BNeV, BNoV, BooV, BParV, BToV, CSpV1, and hunnivirus) were identified in calves with diarrhea using high-throughput sequencing. Bovine astrovirus (genus *Mamastrovirus*, family *Astroviridae*) has been associated with several clinical signs in cattle, including diarrhea and encephalitis [21–23]. In this study, the sequences of BAstV were clustered into groups 2, 4, and 5. Furthermore, group 1 BAstV was previously discovered in Hanwoo cattle with non-suppurative meningoencephalitis in the ROK [24], suggesting that at least 4 distinct groups of BAstV have been identified in the ROK.

Bovine enterovirus (genus *Enterovirus*, family *Picornaviridae*) has been reported in association with diarrhea [25]. The genus *Enterovirus* is classified into a total of 12 species, and BEV is known to belong to species *Enterovirus* E and F [26]. Further studies are needed to determine the clinical relevance of these findings, as there have been reports of BEV causing both diarrhea and respiratory symptoms in cattle [27–29].

Bovine kobuvirus (species *Aichivirus* B, genus *Kobuvirus*, family *Picornaviridae*) is known to cause calf diarrhea [30, 31]. *Aichivirus* strains are classified into 6 species (*Aichivirus* A to F), and the BKoV sequences in this study were classified into *Aichivirus* B and D. *Aichivirus* B, and D have been detected in cattle and other animals associated with diarrhea [30, 32, 33]. Further studies are needed to investigate the epidemiology, ecology, and role of BKoV in calf diarrhea in the ROK.

Bovine nebovirus (genus *Nebovirus*, family *Caliciviridae*), classified as a new genus in 2010, also causes calf diarrhea [34, 35]. To date, 3 BNeV strains (Newbury-1, Dijon, and Kirklareli) have been identified [36–38]. The BNeV sequence obtained here was classified within the Newbury-1 strain, previously reported in Korea [39]. Given that BNeV has not been extensively studied worldwide and its role in calf diarrhea is often underestimated, ongoing research is essential to improve our understanding.

Bovine norovirus (genus *Norovirus*, family *Caliciviridae*) is a major pathogen associated with calf diarrhea, with viral infections in gnotobiotic calves known to cause clinical disease [40, 41]. Noroviruses are classified into seven genogroups (GI to GVII), and all bovine noroviruses reported to date belong to genogroup GIII [42], which is consistent with the findings of this study. However, as noroviruses are considered to have zoonotic transmission potential, continued surveillance is warranted.

Boosepivirus (genus *Boosepivirus*, family *Picornaviridae*) was first identified in diarrhea from cattle in 2009 in Japan [43] and is currently classified into 3 groups: BooV A, B, and C. Only BooV B was detected in this study. Boosepivirus is a relatively recently discovered virus [43, 44], and according to our previous report, BooV B had the highest infection rate (35.7%) among Hanwoo calves in Korea [45]. However, research on its prevalence and impact remains limited, so further investigation is necessary to better understand the role of BooV in calf diarrhea.

Bovine parechovirus (genus *Parechovirus*, family *Picornaviridae*) was first identified in 2021 in the NCBI Sequence Read Archive [46]. The role of BParV in causing calf diarrhea remains uncertain; however, it is hypothesized to be a contributing factor based on previous findings of its initial detection in the digestive tracts of cattle and isolation from calf diarrhea [46, 47]. The genus *Parechovirus* comprises 6 species, *Parechovirus* A-F [47], but BParV was not included in these species. Considering that BParV was initially identified in the ROK, it is essential to conduct further epidemiological investigations of the pathogen in other countries and examine its association with clinical manifestations.

Bovine torovirus (genus *Torovirus*, family *Tobaniviridae*) is known to cause enteric and respiratory diseases in cattle, particularly affecting young calves, and has been reported worldwide [48, 49]. Based on phylogenetic analysis, the *BToV* sequences obtained in this study were distinctly clustered from those found in other animals. However, further studies are needed, as there have been reports suggesting the possibility of interspecies transmission of torovirus [48, 50].

*Cryptosporidium parvum* virus 1 (genus *Cryspovirus*, family *Partitiviridae*) was first identified in the cytoplasm of sporulated oocysts from North American *C*. *parvum* isolates [51]. Phylogenetic analysis of *CSpV1* dsRNA1 and dsRNA2 in this study revealed a tendency for these sequences to cluster according to their geographical origin, consistent with previous studies conducted in Japan and Korea that identified regional genetic variations [52, 53]. While there have been no reports of *CSpV1* pathogenicity in calf diarrhea, some evidence suggests that CSpV1 may influence the pathogenicity of *C*. *parvum* in mice [54]. Further investigation is required to characterize these genetic divergences and assess potential disparities in pathogenicity.

Hunnivirus (genus *Hunnivirus*, family *Picornaviridae*) has been reported in various animal species, including cattle, rats, and cats [55–57], and is suspected to be associated with calf diarrhea [16, 58]. Further research is necessary to elucidate the role of hunnivirus in these clinical manifestations. Based on phylogenetic analysis, the hunnivirus strains identified in this study were classified as *Hunnivirus* A1, which includes the bovine hunnivirus reported from Hungary. Due to the lack of existing research, the epidemiology, host range, and pathogenicity of hunnivirus remain undetermined.

Traditional diagnostic methods for detecting pathogens in clinical samples rely on prior knowledge of potential infectious agents, making diagnosis challenging when such information is unavailable. However, NGS has emerged as a powerful tool to overcome this limitation. Despite certain challenges, including the high cost of NGS and the risk of errors during experiments and data analysis, numerous studies have validated its effectiveness in identifying previously unknown pathogens that 7 viruses including novel bovine betaretrovirus were detected in the non-suppurative encephalitis in cattle and Schmallenberg virus in blood in cattle [59–61].

In line with this trend, this study reported the nearly complete genomes of BAstV groups 2, 4, and 5, BEV, BKoV, BNoV GIII, and BToV for the first time in diarrheic calves in the ROK. Additionally, the presence and nearly complete genome of hunnivirus were also documented for the first time in this context.

While follow-up studies on the viruses identified in this research have yet to be conducted, these newly identified viruses may represent an unrecognized threat in the ROK. These findings may contribute to the development of effective prevention strategies and facilitate further research in this field, thereby enhancing the understanding of the etiology and epidemiology of this important veterinary health concern.

## Conclusion

In this study, 7 pathogens associated with calf diarrhea were detected by PCR, and the detection rates of BRV, *C*. *parvum*, and *Eimeria* spp. significantly increased with higher fecal scores. Additionally, using high-throughput sequencing, the nearly complete genomic sequences of BAstV, BEV, BKoV, BNeV, BNoV, BooV, BParV, BToV, CSpV1, and hunnivirus were identified. This study represents the first investigation to identify the presence of hunnivirus and presents a detailed characterization of the nearly complete genomes of ten viruses associated with calf diarrhea in the ROK. Further research should be needed to understand their impacts on calf diarrhea in Korea, the findings of this study contribute to a better understanding of the epidemiology and molecular characteristics of calf diarrhea-associated pathogens in the ROK.

## Methods

### Sample collection

**Fig. 5:**
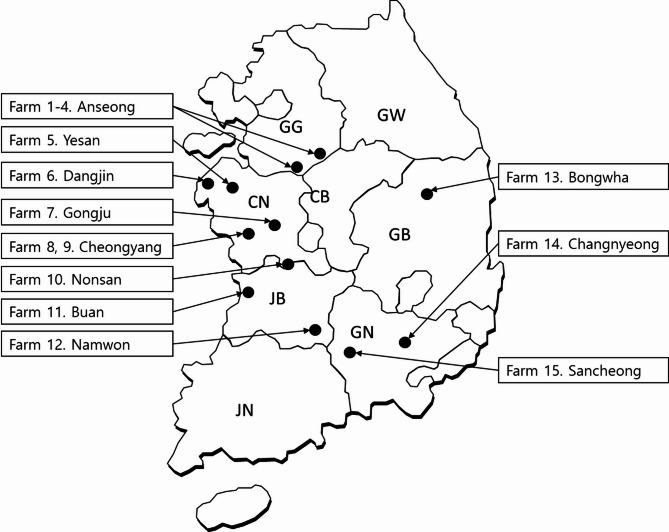
Location of farms included in this study. GG: Gyeonggi-do; GW: Gangwon-do; CB: Chungcheongbuk-do; CN: Chungcheongnam-do; GB: Gyeongsangbuk-do; GN: Gyeongsangnam-do; JB: Jeollabuk-do; JN: Jeollanam-do

In this study, fecal samples from Hanwoo calves up to 60 days of age from 15 different farms in the ROK (Fig. 5) were submitted to the Animal Diagnostic Laboratories of Animal Industry Data Korea, Ltd. for disease diagnosis. Fecal samples were collected via digital rectal palpation and scored on a scale of 0 to 3 (0, Normal consistency; 1, Semi-formed; 2, Loose; 3, Watery) according to the fecal scoring system outlined in the calf health scoring guide developed by the University of Wisconsin-Madison School of Veterinary Medicine and applied by field veterinarians [14]. Fecal scores of 2 and 3 were considered indicative of diarrhea. All samples were stored in 50 ml specimen bottles (SPL Life Sciences, Pocheon, Korea) at 4 °C until transported to the laboratory.

### Pathogen detection by polymerase chain reaction

After the fecal samples were transported to the laboratory, they were suspended in 0.01 M phosphate-buffered saline to create 30% fecal homogenates and centrifuged for 1 min at 100 × g. Total nucleic acids were extracted from the supernatant using the MagMAX™ Total Nucleic Acid Isolation Kit (Thermo Fisher Scientific, Waltham, MA), following the manufacturer’s instructions. All extracts were stored at– 70 °C until pathogen detection. Pathogen detection was performed using PCR or real-time PCR, depending on the targeted pathogen. Specific primer/probe sets for each pathogen are detailed in Table 5 [62–66].

To detect BRV and BCV, RT-PCR was performed using the PrimeScript™ One-Step RT-PCR Kit Ver. 2 (Takara Bio Inc., Shiga, Japan), according to the manufacturer’s protocols, in a 25 µl reaction volume using 2 µl of the extracted template and 23 µl of the reaction mixture. The mixture contained 1 µl of PrimeScript 1-Step Enzyme Mix, 12.5 µl of 2 × 1-Step Buffer, 1 µl of the primer mixture, and 8.5 µl of RNase-Free dH_2_O. The final primer concentration was 0.4 µM. RT-PCR was conducted at 50 °C for 30 min, 94 °C for 2 min, followed by 35 cycles of 94 °C for 30 s, 50 °C for 30 s, 72 °C for 1 min, and a final extension at 72 °C for 5 min. All PCR products were separated by gel electrophoresis on a 1.0% agarose gel stained with RedSafe™ nucleic acid staining solution (Intron Biotechnology Inc., Seongnam, Korea) and subjected to direct sequencing using a dideoxy termination method with an automatic sequencer (3730xl capillary DNA Analyzer; Applied Biosystems, Foster City, CA).

Real-time PCRs were performed using specific primer/probe sets for each pathogen (BVDV 1, BVDV2, *C*. *parvum*, *Eimeria* spp., *Giardia* spp.) (Table 5). Real-time PCR was conducted with the GoTaq One-Step RT-qPCR System (Promega, Madison, WI) following the manufacturer’s recommended protocols in a 10 µl reaction volume using 2 µl of the extracted template and 8 µl of the reaction mixture. The reaction mixture contained 5 µl of GoTaq qPCR Master Mix 2X, 0.2 µl of GoScript™ RT Mix for 1-Step RT-qPCR 50X, 2 µl of the primer and probe mixture, and 0.8 µl of RNase-Free dH_2_O. The final concentrations of primer and probe were 0.3 µM and 0.2 µM, respectively. Real-time PCR was performed using the CFX Opus 96 Real-Time PCR System (Applied Biosystems, Foster City, CA). Cycling conditions were as follows: (a) reverse transcription for 10 min at 45 °C (omitted for *C*. *parvum*, *Eimeria* spp., and *Giardia* spp. PCR); (b) a 10 min activation step at 95 °C; and (c) 40 cycles of 15 s at 95 °C and 60 s at 60 °C. Samples with cycle threshold values less than 35 for targets were considered positive after 40 cycles of the reaction.

**Table 5:** Nucleotide sequences of polymerase chain reaction (PCR) primers/probes for pathogens associated with calf diarrhea

Classifications	Microbial agents	PCR primers, probes	5’- nucleotide sequences − 3’	References
Viruses (conventional PCR)	bovine coronavirus	Forward	CTA GTA ACC AGG CTG ATG TCA ATA CC	[66]
		Reverse	GGC GGA AAC CTA GTC GGA ATA	
	bovine rotavirus	Forward	TCA ACA TGG ATG TCC TGT ATT CCT	[64]
		Reverse	TCC CCC AGT TTG GAA TTC ATT	
Viruses (real-time PCR)	bovine viral diarrhea virus	Forward	CTC GAG ATG CCA TGT GGA C	[63]
		Reverse	CTC CAT GTG CCA TGT ACA GCA	
		BVD type 1 - Probe (FAM/BHQ1)	CAG CCT GAT AGG GTG CTG CAG AGG C	
		BVD type 2 - Probe (HEX/BHQ1)	CAC AGC CTG ATA GGG TGT AGC AGA GAC CTG	
Protozoa (real-time PCR)	*Cryptosporidium parvum*	Forward	CAA ATT GAT ACC GTT TGT CCT TCT GT	[62]
		Reverse	GGC ATG TCG ATT CTA ATT CAG CT	
		Probe (HEX/BHQ1)	TGC CAT ACA TTG TTG TCC TGA CAA ATT GAA	
	*Giardia* spp.	Forward	CAT CCG CGA GGA GGT CAA	[62]
		Reverse	GCA GCC ATG GTG TCG ATC T	
		Probe (FAM/BHQ1)	AAG TCC GCC GAC AAC ATG TAC CTA ACG A	
	*Eimeria* spp.	Forward	AAA GGA TGC AAA AGT CGT AAC AC	[65]
		Reverse	TGC AAT TCA CAA TGC GTA TCG	
		Probe (FAM/BHQ2)	TGT TTC TAC CCA CTA CAT CCA AC	

### Pathogen detection by NGS

Total RNA concentration was measured using Quant-IT RiboGreen (Invitrogen, Carlsbad, CA). To assess RNA integrity, samples were run on a TapeStation RNA ScreenTape (Agilent, Santa Clara, CA). A library was independently prepared with 0.5 µg of total RNA for each sample using the Illumina Stranded Total RNA Library Prep with Ribo-Zero Plus (Illumina, Inc., San Diego, CA). The first step in the workflow involved the removal of rRNA from the total RNA. Subsequently, the remaining mRNA was fragmented into small pieces using divalent cations at elevated temperatures. The cleaved RNA fragments were reverse-transcribed into first-strand cDNA using SuperScript II reverse transcriptase (Invitrogen, Carlsbad, CA) and random primers. This was followed by the synthesis of second-strand cDNA using DNA Polymerase I, RNase H, and dUTP. Next, the cDNA fragments underwent end repair, which included the addition of a single ‘A’ base and ligation of the adapters. The products were purified and enriched by PCR to create a final cDNA library. The libraries were quantified using KAPA Library Quantification kits for Illumina sequencing platforms, following the qPCR Quantification Protocol Guide (KAPA BIOSYSTEMS, Woburn, MA), and qualified using TapeStation D1000 ScreenTape (Agilent Technologies, Santa Clara, CA). Indexed libraries were then submitted to the Illumina NovaSeq (Illumina, Inc., San Diego, CA) for paired-end sequencing (2 × 150 bp). The methodology for metagenomic analysis of viral pathogen detection is described below. Quality control and trimming of short reads were performed using Trim Galore (v.0.6.1) with a Q30 threshold, followed by extraction of viral reads from the dataset using Deconseq (v0.4.3) with 70% query coverage and 90% identity. Subsequently, the viral reads were assembled using the SPAdes assembler (v.3.15.1), and the assembled contigs were annotated using BLAST+ (v.2.10.1) against the NCBI viral database with a rank1 cutoff and an e-value threshold of 1 × e-10. Next, alignment coverage was calculated with the genome of each viral species, and contigs covering 90% of the full sequences of the identified viruses were analyzed.

### Phylogenetic analysis

The obtained sequences were subjected to a BLAST search in the NCBI database. The sequences were aligned using Clustal X (Version 2.0) and examined with a similarity matrix. Phylogenetic analysis was performed using the neighbor-joining method based on nucleotide alignments. Bootstrap analysis was conducted with 1,000 replicates using MEGA version X.

### Statistical analysis

All statistical analyses were performed using SPSS (Version 25.0; IBM Corp., Armonk, NY). The PCR results for each pathogen were recorded as positive or negative and categorized based on fecal scores. The association between fecal scores and each pathogen was compared using Pearson’s χ² test and linear-by-linear association.

## Electronic supplementary material

Below is the link to the electronic supplementary material.

Supplementary Material 1: Supplementary table. The nucleotide identities of the obtained sequences for 10 viruses. These tables include the analysis of full genome sequences for the 10 viruses detected in this study, compared with reported full genome sequences of these viruses.

## Data Availability

The data supporting the findings of this study are available from the corresponding author upon reasonable request. All obtained sequences in this study have already been deposited in the NCBI database.

## Abbreviations

BAstV: Bovine astrovirus
BCV: Bovine coronavirus
BEV: Bovine enterovirus
BKoV: Bovine kobuvirus
BNeV: Bovine nebovirus
BNoV: Bovine norovirus
BooV: Bovine boosepivirus B
BParV: Bovine parechovirus
BRV: Bovine rotavirus
BToV: Bovine torovirus
BVDV: Bovine viral diarrhea virus
CSpV1: *Cryptosporidium parvum* virus 1
NGS: Next-generation sequencing
PCR: Polymerase chain reaction
RdRp: RNA-dependent RNA polymerase
ROK: Republic of Korea
