# Supplementary material for: Molecular characterization of ten viral pathogens causing calf diarrhea in Hanwoo (Bos Taurus coreanae) by next generation sequencing
Source: BMC Vet Res. 2025 Jul 14;21:464. doi: 10.1186/s12917-025-04926-2 (PMC12257796; doi:10.1186/s12917-025-04926-2)
Supplement: Supplementary file 1 — Supplementary Material 1: Supplementary table. The nucleotide identities of the obtained sequences for 10 viruses. These tables include the analysis of full genome sequences for the 10 viruses detected in this study, compared with reported full genome sequences of these viruses. [file 12917_2025_4926_MOESM1_ESM.docx]

**Table S1. The nucleotide identities of the obtained sequences of bovine astrovirus compared to bovine astrovirus group 2 isolate (LC047800)**

| **Sample IDs** | **Nucleotide identities (%)** | | |
| --- | --- | --- | --- |
|  | **Complete genome** | **ORF1ab** | **ORF2** |
| 12151 | 73.8 | 87.34 | 51.45 |
| 18707 | 47.9 | - | - |
| 53954 | - | - | - |
| 71346 | 49.3 | - | - |
| 73961 | - | - | - |
| 86599 | 48.5 | - | - |
| NA_4_475 | 49.2 | - | - |

-, Nucleotide similarity less than 25%.

**Table S2. The nucleotide identities of the obtained sequences of bovine astrovirus compared to bovine astrovirus group 4 isolate (NC_037655)**

| **Sample IDs** | **Nucleotide identities (%)** | | |
| --- | --- | --- | --- |
|  | **Complete genome** | **ORF1ab** | **ORF2** |
| 12151 | 47.4 | - | - |
| 18707 | 60.0 | 71.0 | 46.4 |
| 53954 | 60.2 | 71.0 | 46.9 |
| 71346 | 59.3 | 70.2 | - |
| 73961 | 73.2 | 88.8 | 49.2 |
| 86599 | 61.4 | 71.7 | 49.0 |
| NA_4_475 | 59.6 | 70.6 | - |

-, Nucleotide similarity less than 25%.

**Table S3. The nucleotide identities of the obtained sequences of bovine astrovirus compared to bovine astrovirus group 5 isolate (LC047788)**

| **Sample IDs** | **Nucleotide identities (%)** | | |
| --- | --- | --- | --- |
|  | **Complete genome** | **ORF1ab** | **ORF2** |
| 12151 | 47.8 | - | - |
| 18707 | 78.7 | 84.8 | 68.9 |
| 53954 | 79.3 | 86.6 | 67.1 |
| 71346 | 78.1 | 85.1 | 66.6 |
| 73961 | 60.4 | 71.3 | - |
| 86599 | 82.9 | 90.9 | 70.5 |
| NA_4_475 | 77.6 | 84.3 | 66.7 |

-, Nucleotide similarity less than 25%.

**Table S4. The nucleotide identities of the obtained sequences of bovine enterovirus compared to bovine enterovirus isolate (NC_021220)**

| **Sample IDs** | **Nucleotide identities (%)** | | | | | | | | | | |
| --- | --- | --- | --- | --- | --- | --- | --- | --- | --- | --- | --- |
|  | **Complete genome** | **VP4** | **VP2** | **VP3** | **VP1** | **2A** | **2B** | **2C** | **3A** | **3C** | **RdRp** |
| 18897 | 79.8 | 80.7 | 73.7 | 74.1 | 67.9 | 70.0 | 77.1 | 83.3 | 79.0 | 84.3 | 88.1 |
| 53954 | 76.4 | 75.4 | 75.0 | 75.0 | 57.7 | 74.0 | 79.1 | 81.0 | 79.0 | 85.4 | 86.5 |

**Table S5. The nucleotide identities of the obtained sequences of bovine kobuvirus compared to Aichivirus B isolate (KT003671)**

| **Sample IDs** | **Nucleotide identities (%)** | | | | | | | | | | | |
| --- | --- | --- | --- | --- | --- | --- | --- | --- | --- | --- | --- | --- |
|  | **Complete genome** | **L** | **VP0** | **VP3** | **VP1** | **2A** | **2B** | **2C** | **3A** | **3B** | **3C** | **3D** |
| 00276 | 90.0 | 86.6 | 90.0 | 87.0 | 87.4 | 91.3 | 88.3 | 91.8 | 90.8 | 96.7 | 90.3 | 93.3 |
| 18897 | 89.9 | 86.5 | 89.1 | 88.6 | 87.3 | 92.5 | 87.7 | 93.2 | 90.1 | 85.6 | 92.5 | 92.3 |
| 23358 | - | - | 55.2 | 63.0 | 50.4 | 53.9 | - | 58.7 | - | - | - | 64.9 |
| 71346 | 90.1 | 86.3 | 88.6 | 88.3 | 86.5 | 92.0 | 87.5 | 92.7 | 92.2 | 96.7 | 91.7 | 92.3 |
| 85282 | 90.2 | 85.9 | 89.1 | 88.6 | 87.6 | 82.0 | 87.7 | 93.2 | 90.1 | 95.6 | 52.5 | 92.1 |
| NA_4_475 | 90.2 | 86.1 | 89.2 | 88.8 | 87.6 | 92.3 | 87.7 | 93.2 | 90.1 | 95.6 | 92.4 | 92.1 |
| 53954 | - | - | 57.5 | 59.5 | 54.4 | 55.8 | - | 59.5 | - | - | - | 62.0 |

-, Nucleotide similarity less than 25%.

**Table S6. The nucleotide identities of the obtained sequences of bovine kobuvirus compared to Aichivirus D isolate (LC055960)**

| **Sample IDs** | **Nucleotide identities (%)** | | | | | | | | | | | |
| --- | --- | --- | --- | --- | --- | --- | --- | --- | --- | --- | --- | --- |
|  | **Complete genome** | **L** | **VP0** | **VP3** | **VP1** | **2A** | **2B** | **2C** | **3A** | **3B** | **3C** | **3D** |
| 00276 | - | - | 57.5 | 60.7 | 54.1 | 56.9 | - | 57.0 | - | - | 55.2 | 65.4 |
| 18897 | - | - | 57.1 | 61.9 | 54.2 | 57.9 | - | 58.3 | - | - | 55.9 | 65.4 |
| 23358 | 79.4 | 82.7 | 64.0 | 70.3 | 62.1 | 94.3 | 81.7 | 81.2 | 93.0 | 85.4 | 85.5 | 91.3 |
| 71346 | - | - | 56.3 | 62.0 | 54.1 | 55.9 | - | 57.9 | - | - | 56.1 | 65.1 |
| 85282 | - | - | 57.3 | 61.9 | 54.2 | 57.9 | - | 58.3 | - | - | 55.9 | 65.4 |
| NA_4_475 | - | - | 57.3 | 61.9 | 54.2 | 58.1 | - | 58.3 | - | - | 55.7 | 65.4 |
| 53954 | 83.6 | 85.8 | 82.0 | 74.6 | 75.3 | 92.1 | 79.1 | 81.9 | 91.2 | 83.3 | 84.2 | 90.8 |

-, Nucleotide similarity less than 25%.

**Table S7. The nucleotide identities of the obtained sequences of bovine nebovirus compared to bovine nebovirus isolate (NC007916)**

| **Sample IDs** | **Nucleotide identities (%)** | | |
| --- | --- | --- | --- |
|  | **Complete genome** | **polyprotein** | **ORF2** |
| 83561 | 81.5 | 81.6 | 87.5 |

**Table S8. The nucleotide identities of the obtained sequences of bovine norovirus compared to norovirus GIII type isolate (NC_029645)**

| **Sample IDs** | **Nucleotide identities (%)** | | | | | | | | |
| --- | --- | --- | --- | --- | --- | --- | --- | --- | --- |
|  | **Complete genome** | **p48** | **NTPase** | **p22** | **Vpg** | **Pro** | **RdRp** | **VP1** | **VP2** |
| 73961 | 85.8 | 84.1 | 85.3 | 85.3 | 87.4 | 87.3 | 88.1 | 89.9 | 87.0 |
| 83561 | 85.8 | 83.5 | 85.8 | 85.1 | 86.1 | 87.5 | 88 | 85.9 | 85.3 |

**Table S9. The nucleotide identities of the obtained sequences of boosepivirus compared to boosepivirus B isolate (LC036579)**

| **Sample IDs** | **Nucleotide identities (%)** | | | | | | | | | | | |
| --- | --- | --- | --- | --- | --- | --- | --- | --- | --- | --- | --- | --- |
|  | **Complete genome** | **L** | **VP4** | **VP2** | **VP3** | **VP1** | **2A** | **2B** | **2C** | **3A** | **3C** | **3D** |
| 00217 | 87.3 | 89.6 | 86.1 | 85.0 | 87.6 | 85.1 | 87.9 | 86.7 | 87.5 | 90.0 | 88.6 | 88.6 |
| 00276 | 87.1 | 89.1 | 86.1 | 85.1 | 87.6 | 85.4 | 87.3 | 86.7 | 87.4 | 90.0 | 88.4 | 88.6 |
| 18707 | 86.8 | 90.7 | 86.6 | 85.1 | 88.4 | 83.6 | 87.6 | 85.8 | 86.6 | 86.5 | 89.1 | 87.8 |
| 18897 | 83.0 | 88.0 | 75.1 | 77.6 | 80.5 | 72.7 | 85.2 | 83.6 | 83.7 | 86.8 | 88.6 | 87.7 |
| 23358 | 86.8 | 88.5 | 87.6 | 83.8 | 87.9 | 84.5 | 87.3 | 85.2 | 87.4 | 87.6 | 88.8 | 87.7 |
| 53954 | 87.2 | 89.6 | 86.1 | 85.0 | 87.3 | 85.6 | 87.6 | 86.7 | 87.4 | 90.0 | 88.6 | 88.6 |
| 71346 | 86.8 | 88.5 | 83.1 | 85.5 | 86.3 | 84.6 | 86.7 | 87.3 | 85.0 | 90.2 | 88.8 | 88.9 |
| 83561 | 83.9 | 86.9 | 77.1 | 77.2 | 78.3 | 74.1 | 86.5 | 84.6 | 85.7 | 90.2 | 89.9 | 88.3 |
| 88359 | 83.3 | 89.1 | 79.1 | 78.6 | 82.7 | 73.1 | 84.3 | 85.2 | 85.8 | 87.3 | 89.0 | 87.0 |
| NA_4_475 | 86.0 | 89.6 | 84.6 | 84.1 | 86.7 | 84.8 | 86.5 | 83.6 | 83.8 | 86.2 | 88.6 | 87.8 |

**Table S10. The nucleotide identities of the obtained sequences of bovine parechovirus compared to bovine parechovirus isolate (BR001751)**

| **Sample IDs** | **Nucleotide identities (%)** | |
| --- | --- | --- |
|  | **Complete genome** | **Polyprotein** |
| 18897 | 86.3 | 88.4 |

**Table S11. The nucleotide identities of the obtained sequences of bovine torovirus compared to bovine torovirus isolate (AY427798)**

| **Sample IDs** | **Nucleotide identities (%)** | | | | | |
| --- | --- | --- | --- | --- | --- | --- |
|  | **Complete genome** | **pol1ab** | **S** | **M** | **HE** | **N** |
| 12151 | 80.3 | 78.9 | 95.5 | 94.3 | 87.1 | 69.8 |
| 37284 | 82.1 | 78.9 | 95.5 | 94.3 | 87.1 | 69.8 |

**Table S12. The nucleotide identities of the obtained sequences of dsRNA1 segment of *Cryptosporidium parvum* virus 1 compared to other reported sequence (NC_038843)**

| **Sample IDs** | **Nucleotide identities (%)** |
| --- | --- |
|  | **dsRNA1 (RdRp)** |
| 00245 | 95.8 |
| 00562 | 95.6 |
| 18707 | 96.1 |
| 71346 | 95.8 |
| 85282 | 95.8 |
| NA_4_516 | 96.1 |

**Table S13. The nucleotide identities of the obtained sequences of dsRNA2 segment of *Cryptosporidium parvum* virus 1 compared to other reported sequence (NC_038844)**

| **Sample IDs** | **Nucleotide identities (%)** |
| --- | --- |
|  | **dsRNA2 (capsid protein)** |
| 00245 | 97.8 |
| 00562 | 98.1 |
| 18707 | 97.8 |
| 71346 | 97.8 |
| 85282 | 98.0 |
| NA_4_516 | 98.0 |

**Table S14. The nucleotide identities of the obtained sequences of hunnivirus compared to hunnivirus isolate (NC_018668)**

| **Sample IDs** | **Nucleotide identities (%)** | | | | | | | | | | | |
| --- | --- | --- | --- | --- | --- | --- | --- | --- | --- | --- | --- | --- |
|  | **Complete genome** | **L** | **VP4** | **VP2** | **VP3** | **VP1** | **2B** | **2C** | **3A** | **3B** | **3C** | **3D** |
| 18897 | 81.3 | 87.3 | 84.8 | 65.4 | 69.1 | 57.1 | 85.6 | 87.5 | 82.6 | 84.0 | 86.5 | 90.1 |
| 23358 | 83.9 | 84.1 | 81.5 | 75.0 | 77.7 | 71.2 | 84.9 | 87.0 | 85.0 | 86.4 | 85.3 | 90.6 |
| 85282 | 81.2 | 87.3 | 83.1 | 65.7 | 70.4 | 59.6 | 82.1 | 88.1 | 84.4 | 85.2 | 86.5 | 90.3 |
